# Job Satisfaction and Alcohol Consumption: Empirical Evidence from China

**DOI:** 10.3390/ijerph19020933

**Published:** 2022-01-14

**Authors:** Yuna Ma, Jiafeng Gu, Ruixi Lv

**Affiliations:** 1 Department of Social Work, School of Social Work, China Youth University for Political Sciences, Beijing 100091, China; mayuna@cyu.edu.cn; 2 Institute of Social Science Survey, Peking University, Beijing 100871, China; 3 Department of Applied Mathematics, School of Science, Nanjing Forestry University, Nanjing 210042, China; lvruixi@njfu.edu.cn

**Keywords:** job satisfaction, alcohol consumption, working, logistic regression, China

## Abstract

Despite growing attention to job satisfaction as a social determinant of alcohol-related behaviors, few studies focus on its diverse impacts on alcohol consumption. Using data from the China Family Panel Study in 2018, this study uses logistic regression analysis to examine how job satisfaction affects alcohol consumption in China, finding that people who were satisfied with their jobs were more likely to be regularly drinking. Employed people who were satisfied with their working environment and working hours were more likely to regularly drink, but those who were satisfied with their wages and working security were less likely to be regularly drinking. Findings suggest that the link between job satisfaction and alcohol consumption is dynamic. Employment policies, working wellbeing improvement programs, and alcohol policy improvement should, therefore, be designed on the basis of a comprehensive account of entire job-related attitudes.

## 1. Introduction

Job satisfaction and alcohol consumption are broadly interesting to drinking-behavior researchers, policymakers, and the general public [1,2,3]. An increasing number of studies show that job satisfaction is a fundamental social determinant of alcohol consumption [4,5]. Job satisfaction affects the frequency and quantity of alcohol use [6]. Negative attitudes towards work are generally related to alcohol consumption and alcoholism [1], despite a few being inversely related to alcohol-prone behaviors [7]. However, due to the complexity of job satisfaction and the diversity of working conditions, the impact mechanism of job satisfaction on alcohol consumption has not been clarified, and a general consensus has yet to be reached. Job satisfaction has different dimensions [8]. The majority of academic research examines job satisfaction as a global, single-faceted construct [9,10]. However, the use of a global measure of job satisfaction fails to provide an accurate and full assessment of satisfaction, and it provides little information that management requires, concerning specific aspects of the work environment that employees find satisfying [11]. Therefore, some studies have examined job satisfaction with multi-dimensional scales [8,12]. However, research on the relationship between job satisfaction, with multi-dimensional scales, and drinking is still lacking. The relationship between job satisfaction and alcohol consumption is, by no means, clear at a glance.

The aim of this study was to determine the connections between people’s attitudes towards work characteristics and their alcohol use from a sociological perspective. Cognition about the connections between certain job satisfaction and alcohol consumption can be crucial for employment programming for higher quality of working life and healthier behaviors [13]. As the largest developing country, China has the largest labor market in the world, and the development of working and employment conditions is during a time of economic globalization when an increase in work intensity, job instability, and income inequality is witnessed [14]. In recent years, China has actively promoted a number of employment initiatives to improve people’s wellbeing in the workplace. China is also facing great challenges in controlling the excessive consumption of alcohol [15,16,17]. Thus, China is a very important area for research on job-related attitudes and alcohol consumption behaviors. This study used data from the China Family Panel Study in 2018 to examine the impact of job satisfaction on residents’ alcohol consumption and to determine the impact mechanism. 

The rest of this paper is arranged as follows: Section 2 presents the literature review and hypothesis development; Section 3 presents the data, samples, and methods; Section 4 presents the results; Section 5 is the discussion; Section 6 is the conclusion.

## 2. Literature Review and Hypothesis Development

Quality of working life is often associated with alcohol consumption [18]. Job satisfaction factors, such as rate of pay, hours of work, job security, physical working conditions, and industrial relations are closely relevant to the quality of working life [19,20]. People who are dissatisfied with their job are more likely to have mental-health disorders and behaviors such as problem drinking [21]. Therefore, various national governments and international organizations are actively reducing risky drinking behaviors by improving job satisfaction [22,23]. However, a large gap still exists in our knowledge of the links and pathways between job satisfaction and alcohol use [24]. Research on job satisfaction and alcohol consumption in Western countries is relatively conflicting, having found both a positive and a negative impact of job satisfaction on alcohol consumption [7]. There is still a lack of research in this area in China.

Job satisfaction has different dimensions [8]. Therefore, the overall relationship between job satisfaction and alcohol consumption, and the relationship between alcohol consumption and job satisfaction, in different dimensions, need to be explored [25]. This is the basic logic of this research. On the basis of previous studies [8,26], job satisfaction was divided into four dimensions: working-hour satisfaction, working-environment satisfaction, wage satisfaction, and working-security satisfaction. Among them, working-hour satisfaction emphasizes labor’s perception of time, working-environment satisfaction and working-security satisfaction emphasize labor’s perception of space, and wage satisfaction emphasizes labor’s perception of society. Therefore, these four dimensions are the study of labor’s subjective evaluation of work from the perspective of social space–time. Examining the relationship between job satisfaction and drinking behavior from the overall level and from different dimensions can help to systematically explain the influence of the personal perception of work characteristics on drinking behavior.

Overall job satisfaction and its relationship with alcohol consumption are complex and not fully understood. Job satisfaction is the degree to which a person is satisfied with a number of different features of the job, and overall job satisfaction is the sum of separate satisfaction items derived from different job factors regarding rate of pay, hours of work, job security, physical working conditions, and industrial relations [19,27]. According to Western research, those who have a negative attitude towards their work are also generally alcohol-prone because they are seeking solace in alcohol [1,7]. However, little attention is paid to the relationship between overall job satisfaction and alcohol consumption. Researchers in China currently generally agree that lower levels of satisfaction with life and work are primarily associated with alcohol abuse and alcoholism [28]. In Chinese cultural patterns, drinking is a part of social practice, and social interaction plays the most important role in all drinking factors [15,16]. Features of overall job satisfaction provide social space–time conditions for social drinking. Chinese people who are overall satisfied with their work are more likely to enjoy life and leisure through social drinking [29]. Therefore, the alcohol consumption of people who are satisfied with their job may be higher than that of people who are dissatisfied with their job. Therefore, the following hypothesis is proposed:

**Hypothesis** **1** **(H1).** *People with higher levels of overall job satisfaction have higher alcoholic intake than those with lower levels of overall job satisfaction.*

Working-hour satisfaction is relevant to the quality of working life and health behaviors [30]. Standard working hours are now a global concern because of the established positive alcohol consumption impact associated with long working hours [31]. Exposure to working 41–48 h/week, compared with working 35–40 h /week, may lead to more alcohol consumption [32]. Individuals whose working hours exceed standard recommendations are more likely to increase their alcohol use to levels that pose a health risk, and there was no difference in these associations between men and women or by age, socioeconomic group, and geographical region [33]. Given the fast economic growth in China, Chinese employees experience increased workload and suffer from high levels of time pressure [34]. Research shows that long working hours significantly reduce working-hour satisfaction in China [35]. Therefore, the following hypothesis is proposed:

**Hypothesis** **2** **(H2).** *People with higher levels of working-hour satisfaction have lower alcoholic intake than those with lower levels of working-hour satisfaction.*

Working-environment satisfaction influences work quality, which has attracted the attention of scholars [36,37]. However, little attention has been paid to investigating its impact on drinking behaviors. The working environment includes the physical work environment, human work environment, and organizational environment [38]. With the improvement in people’s working environments over the past few decades [14], the working-environment satisfaction of the Chinese population has been greatly improved. According to data from the National Survey of Social Attitudes and Social Development of China, urban residents had high levels of satisfaction with their working environment in 2015 [39]. However, there are great regional, gender, and age differences among the working-environment satisfaction of the Chinese population in times of economic globalization and a rapid domestic transition from a state-planning-oriented economy to a market economy. 

There is currently a general consensus on the positive relationship between working environment and employees’ job satisfaction [40]. The environmental factor is also an important influence factor for alcohol consumption [41]. Events in which alcohol plays a large role, such as happy hours, holiday parties, or meals with coworkers or clients, are a common part of organizational life [29,42]. The availability and acceptability of alcohol in the workplace play a particular role in fostering harmful alcohol use (i.e., drinking culture) [43]. The people who have positive attitudes towards their working environment might tend to participate in drinking activities and be affected by the culture of acceptance of drinking within the workplace [43]. Therefore, the following hypothesis is proposed:

**Hypothesis** **3** **(H3).** *People with higher levels of working-environment satisfaction have higher alcoholic intake than those with lower levels of working-environment satisfaction.*

Wage satisfaction is an attitude towards wages, which is determined by wage level and increase, social comparison, the wage system and its perception, working characteristics, and working input [44]. With the deepening of the reform and opening-up in China, the wage system has experienced a transformation from “sharing food from the same big pot” to “more pay for more work”. As a result, wage escalation has been high, and wage disparity has expanded during the past 40 years [45]. With the reform of the wage system, the wage satisfaction differential has been prominent across age, gender, educational level, region, sector, and industries in China [46]. The differential of wage satisfaction leads to a series of consequences for individual and organizational development [47], among which the impact on alcohol behaviors has attracted little scholarly attention. The wage satisfaction of residents is positively associated with their subjective happiness [48]. Previous research showed that subjective happiness is inversely associated with alcohol use [49]. Therefore, the following hypothesis is proposed:

**Hypothesis** **4** **(H4).** *People with higher levels of wage satisfaction have lower alcoholic intake than those with lower levels of wage satisfaction.*

Working-security satisfaction refers to an individual’s subjective experience about working security [50], which is the extent to which an organization provides stable employment for employees [51]. The perceived threat of unemployment involves the potential loss of important financial and social resources [52], which is associated with mental health and hazardous behaviors, such as drinking behaviors. A study about young workers in Europe showed that job-security perception appears to be the most important predictive factor for alcohol consumption compared to other socioeconomic factors under study [53]. According to a survey of 2201 rural–urban migrant workers in China, those who have lower levels of working-security satisfaction have significantly higher alcohol consumption [54]. Therefore, the following hypothesis is proposed:

**Hypothesis** **5** **(H5).** *People with higher levels of working-security satisfaction have lower alcoholic intake than those with lower levels of working-security satisfaction.*

**Figure 1:**
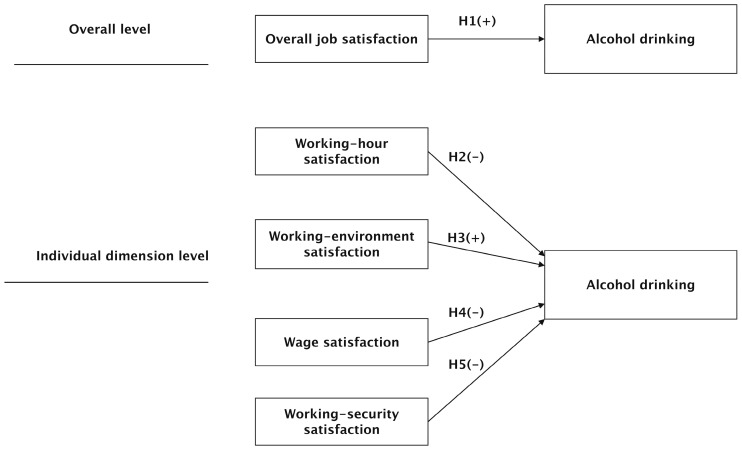
The theoretical framework and related hypotheses.

The theoretical framework and related hypotheses are summarized in Figure 1.

## 3. Methods

### 3.1. Sample

We extracted data used in the study from China Family Panel Studies (CFPS), a comprehensive survey based on individuals, families, and communities by the China Social Science Research Center (ISSS) at Peking University, which are good data for research on drinking behavior [15,16,17]. The CFPS sample was composed of 25 provinces, municipalities, and autonomous regions in the China mainland except for Xinjiang, Tibet, Qinghai, Inner Mongolia, Ningxia, and Hainan. The population of this sample accounted for 95% of mainland China’s population. CFPS mainly conducts face-to-face interviews aided by computer-assisted online interviewing (CAPI). In situations where personal interviews are not feasible, telephone interviews are conducted using computer-assisted telephone interviewing (CATI), or online interviews are conducted using computer-assisted online interviewing (CAWI) to substitute. This study used personal answer questionnaires from the 2018 follow-up study. The dataset included individuals aged 16 or older with full-time working experiences, for a total of 15,657 people interviewed. Afterwards, we deleted 1492 records with a low response rate in the 2018 follow-up survey. The final sample (*n* = 11,547) provided the data for this study.

### 3.2. Measures

#### 3.2.1. Dependent Variables of Alcohol Drinking

Drinking frequency was used to measure alcohol drinking behavior [15,16,17]. “Regularly drink” means more than three times for drinking a week, at least one drink each time. People who tend to become regular drink users are also more likely to become hard drinkers. In the questionnaire, respondents were asked, “In the past month, did you drink more than three times a week?” to obtain the distribution of the independent variable. A positive response was defined as 1, and a negative response was 0.

#### 3.2.2. Working Satisfaction Status

Dissatisfaction with work is more likely to cause mental health issues [21]. People who have psychological conditions are more alcohol-prone [55]. However, some Chinese regard drinking as a way to build their relationships [56]. Thus, there may be two reasons why job satisfaction causes alcoholism. On the one hand, people with higher job satisfaction are more likely to share with friends and therefore, more likely to drink with friends. On the other hand, people with higher job satisfaction are more likely to be content with the status quo and not willing to socialize, and the probability of social drinking is also lower. To constitute the connection between job satisfaction and being alcohol-prone, a binary variable of job satisfaction was generated: satisfying jobs were 1, and other responses were 0. Furthermore, to explore the details, individuals were asked whether they were satisfied with their working security, wages, working environment, and working hours. A positive answer was defined as 1; the opposite was defined as 0. 

#### 3.2.3. Control Variables

Control variables were age, sex, region, Hukou, marital status, education levels, and income. Marital status was divided into two groups: married was defined as 1, and other conditions were defined as 0. Unmarried people were set as the baseline group. Sex was a binary variable: male was defined as 1, and female was defined as 0. We set females as the baseline group. In mainland China, every Chinese citizen’s Hukou status is determined by that of their parents at the time they were born, which is tied to a particular urban or rural location and represents an entitlement to welfare benefits and public services in that place [57]. Hukou was categorized as (1) agricultural and (0) nonagricultural. Nonagricultural was set as the baseline group. Education level was categorized as (1) primary school and below, (2) high school, and (3) 3-year college and above. Primary school and below were set as the baseline group. Regions were categorized as (1) eastern, (2) central, and (3) western regions. The eastern region was defined as the baseline group. Age was categorized as (1) 16–35, (2) 35–50, and (3) 50+. People aged 16–35 were set as the baseline group. Income was categorized as (1) CNY 0–50,000, (2) CNY 50,000–150,000, and (3) CNY 150,000+. Individuals with income of CNY 0–50,000 were set as the baseline group.

### 3.3. Statistical Modeling

After multiple imputations of missing values in the sample (*n* = 11,547), the binary correlation between influencing factors and alcoholism tendencies was described by chi-squared analysis. Data were cleared and processed by StataMP 16. Lastly, we used multivariate logic regression to test each hypothesis, in the theoretical model, through StataMP 16, with *p* values < 0.05 signifying statistical significance.

## 4. Results

Respondents who had working experiences are listed in Table 1, which shows five job satisfaction aspects: wages, working hours, working environment satisfaction, job security satisfaction, overall job satisfaction, and their correlations with drinking. In 2018, 16.6% of respondents drank more than three times a week in the previous month (*n* = 1919), while 83.4% of respondents drank infrequently (*n* = 9628).

Table 1 is a binary cross-table reflecting that the employed people who are more prone to drinking are in central China, male, and married. Significantly, people with agricultural Hukou have obvious tendencies to become alcoholics. Older individuals are also more likely to excessively drink. However, people with higher education levels or lower salaries do not have a strong desire to drink. Those respondents who are unsatisfied with their wages, working time, working security, and working environment have higher drink rates. 

Table 2 presents results of overall job satisfaction and other control variables. People who were satisfied with their jobs were more regularly drinking than those who had employment dissatisfaction (OR: 1.052; 95% CI: 1.006–1.116). Thus, Hypothesis 1 is confirmed. It shows a positive attribution for drinking behaviors in the Chinese, and especially Chinese employment, context. Chinese people who have overall work satisfaction are more likely to enjoy drinking at work and outside of it. It examines the relationship between job satisfaction and alcohol consumption from the influence of an integrated level of job satisfaction in social space–time dimensions. The sum of separate satisfaction items provides a systematic explanation of the influence of the personal perception of work characteristics on their drinking behaviors. The relationship between job satisfaction and alcohol consumption does not change by increasing or decreasing the number of control variables.

Table 3 shows the multivariate logistic regression analysis results with four types of job satisfaction and control variables. Those who were satisfied with their wages were not more regularly drinking than those dissatisfied with their job (OR: 0.923; 95% CI: 0.903–0.945). Thus, Hypothesis 2 is confirmed. Chinese employees who have positive attitudes towards wages might have higher levels of subjective happiness, and less psychological and economic stress, which reduces the possibility of stressful drinking at the workplace. Those who were satisfied with the working environment tended to drink more frequently than those upset with their working environment (OR: 1.034; 95% CI: 1.006–1.062). Thus, Hypothesis 3 is confirmed. Chinese employees who are satisfied with their working environment might have good organizational communication and relationships of interpersonal trust, and increase social interaction and organization activities at the workplace. In this situation, they are probably involved in social drinking activities at the workplace. Those who are satisfied with their working security are not drinking more regularly than those dissatisfied with their working security (OR: 0.909; 95% CI: 0.884–0.934). Thus, Hypothesis 4 is confirmed. Anxiety and depression, resulting from employees’ dissatisfaction with their working security, might increase their drinking, which became more prominent during the COVID-19 pandemic. Those who were satisfied with working hours were more likely to regularly drink than those dissatisfied with their working hours (OR:1.081; 95% CI: 1.054–1.108). Thus, Hypothesis 5 is confirmed. Time pressure in working life is a potential inhibition factor for social drinking. People engaged in long-hour work might spend more time working and not have enough time to participate in drinking activities.

In 2018, employed individuals from eastern regions had more regular drinking than those in the western areas (OR = 0.723; 95% CI: 0.704–0.743/OR = 0.719; 95% CI: 0.647–0.898). People in the central regions were more regularly drinking than those in the eastern regions (OR = 1.068; 95% CI: 1.043–1.094/OR = 1.063; 95% CI: 1.042–1.085). Men tended to more regularly drink than women did (OR = 10.477; 95% CI: 10.141–10.825/OR = 10.574; 95% CI: 10.285–10.870). Furthermore, respondents with agricultural Hukou were more regularly drinking than those with nonagricultural registration (OR = 1.098; 95%CI: 1.071–1.126/OR = 1.098; 95% CI: 1.070–1.116). People aged 36–50 were more regularly drinking than those aged 16–35 (OR: 1.287; 95% CI: 1.250–1.324/OR:1.290; 95% CI: 1.259–1.322). Older individuals aged above 50 drank more excessively than those aged 16–35 (OR: 1.570; 95% CI: 1.522–1.619/OR:1.557; 95% CI: 1.517–1.598). Those with higher education were less likely to regularly drink than those with lower education (OR: 0.498; 95% CI: 0.479–0.518/OR:0.489; 95% CI 0.473–0.506). Respondents with medium-level education were regularly drinking less than those with lower education (OR: 0.862; 95% CI: 0.840–0.884/OR:0.854; 95% CI: 0.836–0.873). Those with total income between CNY 50,000 and 150,000 were more likely to be regularly drinking than those with a total annual income below CNY 50,000 (OR: 1.184; 95% CI: 1.151–1.218/OR:1.171; 95% CI: 1.143–1.199). Individuals with a total income above CNY 150,000 were more regularly drinking than those with an income below CNY 50,000 (OR: 1.040; 95% CI: 1.015–1.066/OR:1.036; 95% CI: 1.015–1.058). Married people had a more obvious tendency to regularly drink than that of other marital-status types (OR: 1.412; 95% CI: 1.370–1.455/OR:1.411 95% CI: 1.375–1.447). The relationship between job satisfaction and alcohol consumption does not change by increasing or decreasing the number of control variables.

## 5. Discussion

There are some studies on the relationship between job satisfaction and alcohol consumption in Western countries [40,58,59]. However, there is a lack of relevant research in China. The results of this study show that the alcohol consumption of people with higher levels of overall job satisfaction is higher than that of people with lower levels of overall job satisfaction. The hypothesis 1 is confirmed. This conclusion is inconsistent with the conclusions of related studies conducted in Western countries. The positive impact of overall job satisfaction on alcohol consumption does not have universality. China is unique in this issue, as it has a deep drinking culture that mainly shows the humanistic dimension, and Chinese people care about the function brought by drinking liquor, while the goal of Western drinking is to appreciate and enjoy alcohol, pursuing enjoyment the flavor in communication [60]. In China, alcohol is frequently regarded as a communication tool. The consumption of alcohol at the workplace is an important communicational tool in working life in China. Research indicated a significant positive relationship between job satisfaction and communication in the workplace in China [56]. A similar situation exists in Arab [61] and Indonesian [62] companies. Therefore, Chinese people who are overall satisfied with their job have more communicational space to enjoy social drinking at the workplace. The positive relationship between overall job satisfaction and alcohol consumption shows that the aggregate of job satisfaction dimensions on working hours, working environment, wages, and working security provides social space–time conditions for alcohol consumption.

Working hours are closely related to job satisfaction and alcohol consumption [33]. As the largest country in the Asia Pacific with rapid industrialization, China is undergoing dramatic economic and social transformation that hugely impacts work, employment, and workers’ experiences of wellbeing. Time pressure at work is increasingly recognized for its adverse impact on levels of employee wellbeing [63]. The 996 work system is a microcosm of the prevalence of overtime work culture in China [64]. The 996 is a work system with an extension of legal working hours originated in Chinese Internet companies. It refers that people go to work at 9 a.m., and leave work at 9 p.m., resting at noon for 1 h (or less), and in the evening for 1 h (or less), which means working for a total of 10 h a day and 6 days a week. This kind of work schedule has attracted much controversy in China.

In modern industrial society, working-hour satisfaction is threatened by overtime work [65]; however, its impact on alcohol consumption has not received sufficient attention. Results of this study show that people with higher levels of working-hour satisfaction have higher alcoholic intake than those with lower levels of working-hour satisfaction. The hypothesis 2 is not confirmed. It appears to be the reverse of what might be expected from Western research, i.e., it might be reasonable to predict that those who were dissatisfied with working hours would seek solace in alcohol to deal with time pressure. However, it may be that those who perceived reasonable working hours would spend more time on socializing, which happens with alcohol in Chinese culture. Those who are dissatisfied with working hours might be suffering from long, inflexible working hours and a lack of enough social time to enjoy drinking. 

Work time is a major shaper of human activities [66], including drinking behaviors. Results of this study show that working-hour satisfaction is a temporal structural factor of job satisfaction that also deeply shapes employees’ drinking behaviors, and it is culture-specific in China. The positive relationship between job satisfaction and alcohol consumption is a result of the interaction between time structures and social structures.

Employees’ attitudes towards the working environment are closely related to alcohol consumption. Results of this study show that people with higher levels of working-environment satisfaction have higher alcoholic intake than those with lower levels of working-environment satisfaction. The hypothesis 3 is confirmed. This is inconsistent with findings of a previous study, stating that, when employees feel content at work, the rate of drinking alcohol daily at work decreases in some Western countries [67]. The different results might come from different drinking cultural patterns. In some Western countries, people who feel discontent with the work environment might increase their daily drinking as a stress-coping mechanism [67]. In China, employees have higher levels of work-environment satisfaction when they are content with the organizational communication and interpersonal trust, such as social interactions, communication space, and organizational activities at the workplace [68], which provides more opportunities and social networking for their social drinking. Chinese employees who are dissatisfied with their working environment might have poor organizational communication and interpersonal trust, and they may actively, or passively, reduce social interaction and organizational activities at workplace. In this situation, they probably work in self-enclosed worlds and are less involved in social drinking. 

Environmental factors are an important influence for alcohol consumption [41]. Results of this study show that working-environment satisfaction, as a spatial structural factor of job satisfaction, also deeply shapes employees’ drinking behaviors, and it is culture-specific in China. The positive relationship between work-environment satisfaction and alcohol consumption is a result of the interaction between spatial and social–cultural structures.

Wage satisfaction connects wage and personal behaviors [69], and its influence on the personal and organizational dimensions is receiving increasing attention [70]. Generally, people are concerned about absolute income, but some studies have recently suggested that people are also concerned about relative income status [71]. That wage satisfaction depends on reference wage is now part of the empirical happiness literature [72]. Employees care about their coworkers’ wages and suffer from disadvantageous comparisons [72]. With the deepening of reform and opening-up in China, wage differences related to gender, educational, regional, and industrial differences have been growing in the past three decades [73], and the resulting wage-satisfaction differences have attracted global academic attention. Results of this study show that people with higher levels of wage satisfaction have lower alcoholic intake than those with lower levels of wage satisfaction. The hypothesis 4 is confirmed.

Wage satisfaction proved to be a negative predictor of drinking, perhaps indicating that those who have negative attitudes towards their wages might want to increase them through moving upward in the workplace or changing jobs, and the related employment resources and opportunities could be facilitated in social activities, carried out with the assistance of alcohol, in China. This explanation is partly consistent with the findings that wage dissatisfaction is positively associated with turnover and absence in China [45]. From another point of view, people who have negative attitudes towards their wages might have significantly low levels of subjective happiness and seek solace in alcohol to cope with the psychological and economic stress [70]. The two kinds of coping mechanisms seem to support this argument, i.e., the less employees are satisfied with wages, the more alcohol they drink.

Perceptions of working insecurity have detrimental effects on job satisfaction and employee wellbeing [74]. The results of this study show that people with higher levels of working-security satisfaction have lower alcoholic intake than those with lower levels of working-security satisfaction. The hypothesis 5 is confirmed.

When people are worried about losing the present job or the high risk of their work, positive impacts on alcohol-prone behaviors are likely. This is consistent with findings of previous studies [3,39,59]. Ensuring working-security satisfaction is currently a major challenge under the influence of the COVID-19 pandemic, which has caused massive unemployment [75]. In the US, COVID-19 has resulted in approximately 13 to 36 million Americans losing employment [75]. A similar situation exists in China. Millions of people suffer from the anxiety and depression resulting from employment loss since the start of the COVID-19 pandemic [59]. Our research further proves the necessity of improving employees’ working-security satisfaction for healthier behaviors. 

In addition, this research shows that the people in the central regions are more alcohol-prone than those in the eastern and western regions. Moreover, this research shows that men tend to drink more frequently than women do; those with an agricultural Hukou are more likely to drink than those with a nonagricultural registration; older people are more alcohol-prone than younger people; those with a high degree of education are less likely to abuse drinking than those with a lower degree; those with a higher income are more alcohol-prone than those with a lower income; those with a spouse are more likely to be hard drinking than those without a spouse. Both individual demographics and socioeconomic factors impact alcohol consumption.

This study contributes to the extant literature in three ways. First, it extends the understanding of the association between job satisfaction and alcohol consumption. Second, it extends the social space–time theory of alcohol use by drawing insights obtained from a daily working lens. Third, different dimensions of job satisfaction were found to influence alcohol consumption. This finding does not coincide with the overwhelming majority of the literature on this topic. The existence of this kind of counterexample shows that the relationship between job satisfaction and alcohol consumption is complex and nonlinear. Therefore, it helps to eliminate people’s mindset and aid people to objectively and comprehensively evaluate the impact of job satisfaction on alcohol consumption. These findings have important practical guiding value for the formulation of relevant policies.

There was an important correlation between job satisfaction and alcohol consumption. However, due to the content of the questionnaire and the limitations of the survey, this research has the following limitations. First, due to the limitations of the data, job satisfaction indicators investigated in this research are limited, and other factors may have been omitted. Second, because of the attributes of cross-sectional data, it is difficult to reveal the causal mechanism of job satisfaction on alcohol consumption. Third, for the measurement of alcohol consumption, this study uses the frequency of weekly drinking and lacked the quantity of alcohol use. In addition, the meaning of job satisfaction is becoming increasingly complex, and there are often interactions with personal characteristics. Thus, the relationship between job satisfaction and alcohol consumption needs further study. This study shows that region, gender, and age factors affect job satisfaction, but the interaction variables of these factors are not introduced into the model. Therefore, it is impossible to examine the interaction effects of these factors. This is worthy of further study in the future.

## 6. Conclusions

This study used CFPS2018 data to empirically study the impact of job satisfaction on alcohol consumption. The following job satisfaction dimensions were found to affect the alcohol consumption of residents: working-environment satisfaction and working-hour satisfaction were associated with higher levels of alcohol consumption; working-security satisfaction and wage satisfaction were associated with lower levels of alcohol consumption. Furthermore, overall job satisfaction was associated with higher levels of alcohol consumption. In addition, region, sex, Hukou, age, education level, income, and marital status all significantly impact the alcohol consumption of the population. These results have implications for employment policies, working wellbeing improvement programs, and alcohol policy improvement for a higher quality of working life and healthier behaviors in China and other countries. It also informs the types of interventions required to address the problem of excessive consumption of alcohol in the field. Strategies that aim to control the excessive consumption of alcohol may do well to focus on a comprehensive account of all job-related attitudes on social space-time dimensions.

## Figures and Tables

**Table 1:** Distribution of general characteristics.

	Number of Participants (*n* = 11,547)		Drinking		$\chi^{2}$ (*p*)
	Classification	N (%)	Yes N (%)	No N (%)	
Overall job satisfaction	Satisfied	7923 (68.6)	1296 (67.5)	6627 (68.9)	1.33 (0.249)
	Others	3624 (31.4)	624 (32.5)	3000 (31.1)	
Wage satisfaction	Satisfied	6795 (58.8)	1106 (57.6)	5689 (59.1)	1.40 (0.237)
	Others	4752 (41.2)	813 (42.4)	3939 (40.9)	
Working-security satisfaction	Satisfied	8783 (76.1)	1376 (71.7)	7407 (76.9)	24.02 *** (0.001)
	Others	2764 (23.9)	543 (28.3)	2221 (23.1)	
Working-environment satisfaction	Satisfied	8062 (69.8)	1282 (66.6)	6780 (70.4)	9.92 ** (0.002)
	Others	3485 (30.2)	637 (30.4)	2848 (29.6)	
Working-hour satisfaction	Satisfied	8055 (69.8)	1336 (69.6)	6719 (69.8)	0.02 (0.885)
	Others	3492 (30.2)	583 (30.4)	2909 (30.2)	
District	Eastern region	5441 (47.1)	909 (48.1)	4452 (46.9)	27.76 *** (0.001)
	Central region	3356 (29.1)	605 (40)	2717 (28.6)	
	Western region	2750 (23.8)	377 (19.9)	2331 (24.5)	
Gender	Male	6514 (56.4)	1725 (91.2)	4694 (49.4)	1100.00 *** (0.001)
	Female	5033 (43.6)	166 (8.8)	4806 (50.6)	
Hukou	Agriculture	7628 (66.1)	1321 (69.9)	6187 (65.1)	15.71 *** (0.001)
	Nonagriculture	3919 (33.9)	570 (30.1)	3313 (34.9)	
Age	16–35	4916 (42.6)	562 (29.7)	4279 (45)	199.77 *** (0.001)
	36–50	3831 (33.2)	663 (35.1)	3114 (32.8)	
	>50	2800 (24.3)	666 (35.2)	2107 (22.2)	
Highest level of education	Primary school and below	2867 (24.8)	579 (30.2)	2288 (23.8)	152.63 *** (0.000)
	High school	5740 (49.7)	1064 (55.4)	4676 (48.6)	
	3-year college and above	2940 (25.5)	276 (14.4)	2664 (27.6)	
Income	CNY 0–50,000	6003 (67.3)	902 (47.7)	5043 (68.3)	18.60 *** (0.001)
	CNY 50–150,000	2220 (24.9)	406 (21.5)	1791 (24.3)	
	>CNY 150,000	3324 (7.8)	583 (8.3)	2666 (28.1)	
Marital status	Married	8721 (75.5)	1583 (83.7)	7031 (74)	80.52 *** (0.001)
	Other	2826 (24.5)	308 (16.3)	2469 (26)	
Regularly drink	Yes	1919 (16.6)			
	No	9628 (83.4)			

Note. ***, *p* < 0.01. **, *p* < 0.05.

**Table 2:** Multivariable logistic regression analysis.

Characteristics	Odds Ratio	Drink_Y_18 95% CI	*p* Value
Work satisfaction			
Satisfied vs. others	1.025	1.006–1.116	0.010
District			
Western vs. eastern	0.719	0.647–0.898	0.001
Central vs. eastern	1.063	1.042–1.085	0.000
Gender: male vs. female	10.574	10.285–10.870	0.000
Hukou: agricultural vs. nonagricultural	1.093	1.070–1.116	0.000
Age group			
36–50 vs. 16–35 years	1.290	1.259–1.322	0.000
50+ vs. 16–35 years	1.557	1.517–1.598	0.000
Education			
High vs. low	0.489	0.473–0.506	0.000
Medium vs. low	0.854	0.836–0.873	0.000
Personal income			
CNY 50,000–149,999 vs. CNY 0–49,999	1.171	1.143–1.199	0.000
CNY 150,000+ vs. CNY 0–49,999	1.036	1.015–1.058	0.001
Marital status: married vs. other	1.411	1.375–1.447	0.000
Constant	0.025	0.024–0.026	0.000
LR chi-squared	57,909.12 (0.000)		
−2Log likelihood	315,358.08		
Cox and Snell R square	0.130		
MacFadden square	0.155		
Nagelkerke square	0.220		

**Table 3:** Multivariable logistic regression analysis.

Characteristics	Odds Ratio	Drink_Y_18 95% CI	*p* Value
Wage satisfaction			
Satisfied vs. others	0.923	0.903–0.945	0.000
Working-environment satisfaction			
Satisfied vs. others	1.034	1.006–1.062	0.016
Working-safety satisfaction			
Satisfied vs. others	0.909	0.884–0.934	0.000
Working-hour satisfaction			
Satisfied vs. others	1.081	1.054–1.108	0.000
District			
Western vs. eastern	0.723	0.704–0.743	0.000
Central vs. eastern	1.068	1.043–1.094	0.000
Sex: male vs. female	10.477	10.141–10.825	0.000
Hukou: agricultural vs. nonagricultural	1.098	1.071–1.126	0.000
Age group			
36–50 vs. 16–35 years	1.287	1.250–1.324	0.000
50+ vs. 16–35 years	1.570	1.522–1.619	0.000
Education			
High vs. low	0.498	0.479–0.518	0.000
Medium vs. low	0.862	0.840–0.884	0.000
Personal income			
CNY 50,000–149,999 vs. CNY 0–49,999	1.184	1.151–1.218	0.000
CNY 150,000+ vs. CNY 0–49,999	1.040	1.015–1.066	0.000
Marital status: married vs. other	1.412	1.370–1.455	0.000
Constant	0.026	0.025–0.028	0.000
LR chi-squared	41,756.03 (0.000)		
−2Log likelihood	227,400.94		
Cox and Snell R square	0.130		
MacFadden square	0.155		
Nagelkerke square	0.220		

## Data Availability

The data for this research can be applied and downloaded from the Peking University Open Research Data Platform website (https://opendata.pku.edu.cn/dataverse/CFPS) (accessed on 25 June 2021).
